# Systematic Review and Meta-Analysis on the Role of Chemotherapy in Advanced and Metastatic Neuroendocrine Tumor (NET)

**DOI:** 10.1371/journal.pone.0158140

**Published:** 2016-06-30

**Authors:** Matthew H. Wong, David L. Chan, Adrian Lee, Bob T. Li, Sumit Lumba, Stephen J. Clarke, Jaswinder Samra, Nick Pavlakis

**Affiliations:** 1 Department of Medical Oncology, Gosford Hospital, Gosford, Australia; 2 Kolling Institute of Medical Research, University of Sydney, Sydney, Australia; 3 Department of Medical Oncology, Royal North Shore Hospital, Sydney, Australia; 4 Northern Cancer Institute, Sydney, Australia; 5 Division of Solid Tumor Oncology, Department of Medicine, Memorial Sloan Kettering Cancer Center, New York, New York, United States of America; 6 Sydney Medical School, University of Sydney, Sydney, Australia; 7 Department of Gastrointestinal Surgery, Royal North Shore and Macquarie University Hospitals, Sydney, Australia; Davidoff Center, ISRAEL

## Abstract

**Background/Objectives:**

In the era of somatostatin analogues and targeted therapies, the role of chemotherapy in NET remains largely undefined. This systematic review aimed to assess the effect of chemotherapy on response rates (RR), progression-free survival (PFS), overall survival (OS) and toxicity compared to other chemotherapies/systemic therapies or best supportive care in patients with advanced or metastatic NET.

**Methods:**

Randomised controlled trials (RCTs) from 1946 to 2015 were identified from MEDLINE, EMBASE, other databases and conference proceedings. Review of abstracts, quality assessment and data abstraction were performed independently by two investigators. Meta-analyses were conducted using Mantel-Haenszel analysis with random-effects modelling.

**Results:**

Six RCTs comparing standard streptozotocin plus 5-fluorouacil (STZ/5FU) chemotherapy to other chemotherapy regimens, and 2 comparing this to interferon (IFN) were included. Only 1 study was considered at low risk of bias. STZ/5-FU was no different to other chemotherapies in response rate [RR 0.96; 95% confidence interval (CI) 0.72–1.27], PFS (RR 0.95; CI 0.81–1.13), or OS (RR 1.03; CI 0.77–1.39). IFN may produce higher response than STZ/5FU (RR 0.20; CI 0.04–1.13), but event rates were small and survival was no different. Interferon was associated with higher overall haematological (RR 0.47; CI 0.27–0.82) and lower overall renal toxicity (RR 3.61; CI 1.24–10.51).

**Conclusion:**

Strong evidence is lacking in the area of chemotherapy in neuroendocrine tumors. There is currently no evidence that one chemotherapeutic regimen is significantly better than the other, nor is interferon better than chemotherapy. There is an urgent need to design RCTs comparing modern chemotherapy to other agents in NET.

## Introduction

### Rationale and objective

Neuroendocrine tumors (NET) are cancers originated from cells with neurotransmitter, neuromodulator or neuropeptide hormone production properties; and they encompass a wide spectrum of diseases including carcinoid or non-carcinoid gastro-enteropancreatic (GEP) tumors, catecholamine secreting tumors and others[1]. While NET remains a rare cancer, its incidence has steadily increased from 1.09/100,000 (1973) to 5.25/100,000 (2004).[2] NET comprises of a wide spectrum of disease. Although the majority of NET is indolent and well-differentiated with a median overall survival (OS) of over 6 years, patients with poorly differentiated tumors have a median OS of only 10 months.[2] Surgery and chemotherapy were the sole treatment options in the 70s to 90s, but nowadays there is a plethora of systemic (somatostatin analogues, targeted therapies, interferon (IFN)) and locoregional therapies (trans-arterial chemo-embolisation, radionuclide therapies, ablation therapies, radiotherapies) that can be offered to these patients.[3] That said, given the rarity of these cancers, there remains few randomised controlled trials in this field, and the evidence of these treatment is therefore considered much weaker than more common cancers.[3] In addition, there is no single accepted standard of care approach for metastatic disease. Although guidelines from international organisation such as ENETS and NANETS have suggested potential treatment algorithms [4–6], treatment can vary widely based on patient factors as well as treatment centres. Even though certain NET subtypes such as pancreatic NET (pNET) appears more sensitive to chemotherapy, chemotherapy generally has modest response rates in NET. Furthermore, the adverse effects of chemotherapy may exceed the efficacy of these drugs.[7,8] Streptozotocin (STZ), an antibiotic with C-2 glucose-substituted methylnitrosourea moiety with alkylating and possible carbamoylating properties, was the first chemotherapy studied in NET.[9] It was first reported to offer meaningful clinical benefits in patients with pNETS.[10,11] Later, this was combined with 5FU in a 5-day intensive regimen, and was shown to be both feasible and effective in a small non-randomised trial.[12] Other chemotherapies that had been studied in NET included dacarbazine (DTIC), cisplatin, etoposide, carboplatin and temozolomide, but most of these were only studied in single arm trial design.[3,8] Currently, STZ-based regimen is considered an acceptable treatment modality for NET, especially those that are well to moderately differentiated and originated from pancreas.[3] However, few systematic reviews have previously been published addressing the role of chemotherapy critically, and meta-analyses has not been performed.[13,14]

The classification of NETs has also evolved over the past few decades as the above trials have been conducted. Whilst initial systems did not include formal grades, the most recent WHO classification system in 2010 provided formal, quantitative cutoffs delineating between the three histological grades. Given its association with clinical aggressiveness and prognosis [15], this has been of great help in selecting more homogeneous cohorts for inclusion in trials. At the same time, development of radiological response criteria such as RECIST helped to standardize the reporting of clinical trials and diminish the amount of measurement bias particularly in open-label trials. Therefore, trials conducted prior to standard criteria are even more susceptible to bias, and careful critical evaluation is essential in order to obtain a fair summary of the efficacy of chemotherapy in NETs.

In light of this, we conducted a systematic to comprehensively search and critically appraise the existing evidence for chemotherapy and to compare its efficacy against other therapies in neuroendocrine tumors.

## Methods

### Eligibility criteria

We included randomised controlled studies of neuroendocrine tumors in adults as per the WHO classification[16], comparing a) chemotherapy with control b) standard chemotherapy with other chemotherapies, and c) standard chemotherapy versus other systemic treatments. Exclusion criteria were studies of other tumors, studies with no chemotherapy, non-interventional studies and non-randomised studies. Primary endpoints were RR and PFS, and secondary endpoints OS and toxicity. This protocol was not prospectively registered online.

### Information sources

We performed systematic searches on the MEDLINE (1946 to current), EMBASE (1974 to 2015), Cochrane Central Register of Controlled Trials, Cochrane database of systematic reviews, ACP journal club and DARE. Bibliography searches were conducted on selected systematic/ narrative reviews identified on literature search. Electronic search of abstracts in search engines of major conferences proceedings on the American Society of Clinical Oncology (ASCO) and European Society of Medical Oncology (ESMO), as well as Journal of Clinical Oncology (JCO) and Annals of Oncology. Where study information was lacking, the sponsors of trials (pharmaceutical companies and/or hospitals) as well as lead authors of protocols were contacted for any details required.

### Search strategy

In electronic database searches we included the MeSH terms and textwords of "chemotherapy" OR "antineoplastic agents" OR "streptozotocin" OR "fluorouracil" OR "doxorubicin" OR "dacarbazine" or "temozolamide" for the search of chemotherapy (intervention); "neuroendocrine tumors" OR "carcinoid tumors" OR "insulinomas" OR "gastrinomas" OR "islet cell carcinoma" OR "APUD tumors" for NET (patients); "randomized controlled trial.pt." OR "controlled clinical trial.pt." OR "randomized.ab." OR "placebo.ab." OR "clinical trials as topic/" OR "randomly.ab." OR "trial.ti." for a sensitive search for randomised trials; and "Review.pt" and "Medline.tw" OR "Meta analysis.pt" OR "systematic$.tw" and "(review$ or overview$).tw" OR "meta?analy$.tw" OR "meta analy$.tw" for systematic/ narrative reviews. All languages published were considered (S1 Table).

### Study selection

Only studies with randomised design were included. Studies with a combination of randomised and direct assignment were only included if results for randomised treatment arms were separately available. Participants of included trials all had progressive or advanced/ metastatic NET. NET was often loosely defined, and many definitions existed for NET. For the purpose of this review, the WHO classification of NET was adopted, which broadly included carcinoid tumors and islet cell tumors. Randomised studies of phaeochromocytoma, merkel cell tumors and were excluded as these malignancies have different clinical courses and prognoses compared to NET. Small cell carcinomas were also excluded as small cell carcinomas have a much more aggressive course compared to well-differentiated NETs, and studies in these tumours accordingly used substantially different treatment modalities (such as platinum-based doublet chemotherapy). All chemotherapy studies were included, regardless if the intervention/ experimental arm was another chemotherapy or other treatment modalities.

### Data extraction and risk of bias assessment

Two investigators (MW, SL) independently reviewed full-text manuscripts of included studies, and extracted data for inclusion and exclusion criteria, baseline characteristics, study design and endpoints. Each study was evaluated for quality assessment based on the Cochrane Collaboration's 'Risk of bias' tool and the PRISMA guide (S1 PRISMA Checklist), with particular focus placed on the domains of randomisation, allocation concealment, blinding, intention-to-treat (ITT) analysis and selective reporting.[17] Where the primary references did not provide sufficient details, we resorted to secondary references, abstracts, presentations or protocols. Studies were rated overall high risk of bias if there were significant issues with randomisation or outcome measurement, giving rise to selection or measurement bias. The outcomes that were abstracted and analysed included response rate, progression-free survival, overall survival and toxicity.

### Summary measures and methods or analysis

The principal summary measure for meta-analysis was risk ratio using the Mantel-Haenszel analysis methods. A random effect model was used since studies included were heterogeneous and varied widely in patient characteristics, treatment arms, follow-up time and years of study accrual. Point estimate of risk ratios and 95% CI (confidence intervals) were provided, along with forest plots. We initially aimed to perform meta-analysis of studies with an overall low risk of bias. However, only one study was assessed as having low risk of bias[18], and thus the primary objective could not be undertaken. A sensitivity analysis combining all trials was performed, although only a conservative conclusion could be made in light of this. Other pre-planned subgroup analyses included were studies with long or short follow up time (> = 4 or <4 years follow up), location of tumors and different chemotherapy regimens. 4-years follow-up time as a cut-off was chosen as the subgroup stratification was chosen since in the phase III study of somtatostatin analogue sandostatin LAR (PROMID) which has one of the longest follow up time, 90% of patients have had a recurrence event by 4 years with minimal drop-offs after this. [19] For a cancer that is relatively indolent, the hypothesis generated was that studies with longer follow up were more likely to identify late recurrence and thus address the null hypothesis. -Where there was missing data, attempts were made to calculate or estimate the outcome measures from existing data or Kaplan-meier curves. Only direct evidence is meta-analysed in the treatment network studied. Heterogeneity was calculated using Cochrane Q (Chi-squared) and I^2^ statistics. If heterogeneity was observed, this was addressed by excluding and including trials, performing pre-planned subgroup analyses and identifying clinical and methodological diversity which might contribute to heterogeneity. If heterogeneity remained substantial and significant despite these methods, the meta-analyses results were disregarded. Publication bias was assessed using standard funnel plots. An asymmetry in the base of the funnel plot (small SE) with RR >1 indicates a paucity of small negative trials, and suggests the possibility of publication bias.

## Results

### Study selection

**Fig 1:**
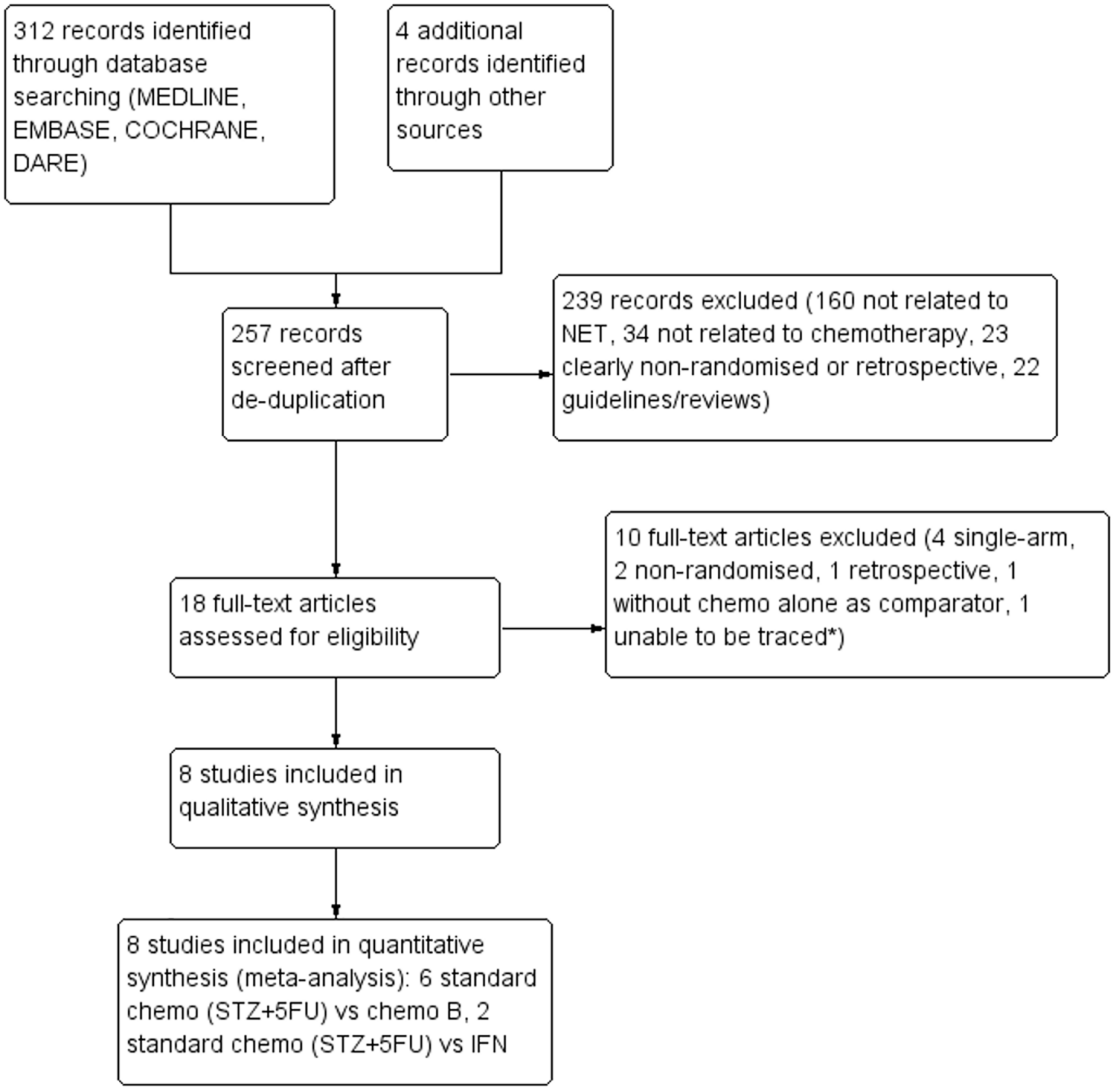
Flow diagram of study selection.

Altogether we identified 312 potentially relevant studies with 261 from the RCT searches and 51 from systematic review searches. Bibliography searches in these narrative and systematic reviews found another 4 potentially relevant RCTs. No new randomised studies (published or unpublished) were identified by hand searches or correspondence with pharmaceutical companies. A systematic review was previously performed on chemotherapy and NET [13], and another was performed for all pharmacotherapies in NET[14]; but in both systematic reviews, a limited search using Pubmed was performed, non-randomised studies were included, and a qualitative review was performed with no meta-analyses. After filtering using pre-defined inclusion and exclusion criteria, 8 studies were included for quality assessment and data extraction (Fig 1).

### Characteristics of included studies

Six studies compared one chemotherapy regimen against another (S2 Table).[20–24] [25] The control regimen was STZ plus 5FU in all studies except from Moertel (1980), where STZ plus 5FU was compared to the previous standard STZ alone.[22] One of these six studies, Meyer (2014), compared STZ plus capecitabine with STZ, capecitabine and cisplatin. Given capecitabine is a prodrug of 5FU, we elected to include this trial in the above analysis with STZ plus capecitabine as the control arm. Aside from age and performance status, most baseline characteristics were heterogeneous or insufficient in these trials. Among the 4 studies with information about location of tumors of patients included in studies, the proportion of patients with mid-gut tumors varied [31% in Sun (2005), 51% in Moertel (1979), 36% with 44% from unknown locations in Engstrom (1984), and 20% with 32% from unknown locations in Meyer (2014)].[20,21,24] [25] These studies also varied in their follow-up time, ranging from 2–9 years.[20–24] Proportion of patients on prior chemotherapy and surgery was only included in 2 studies respectively,[21,24] and there was no information on prior radiotherapy and somatostatins in any of these trials. Two studies compared chemotherapy against interferon (S2 Table).[18,26] The age and ECOG 0/1 proportion were similar between these and the chemotherapy versus chemotherapy trials. However, these 2 trials had much higher proportion of patients with midgut tumors (63% in Dahan (2009); 95% in Oberg (1989)], and they appeared to include patients with more advanced disease, as demonstrated by a very high proportion of patients with progressive disease (99%) and liver metastases (84%) in Dahan (2009), and a 100% rate of active carcinoid symptoms including a 30% rate of heart failure in Oberg (1989).[18,26] No studies were identified comparing chemotherapy and targeted therapy or somatostatin analogues, and no studies compared chemotherapy with best supportive care (BSC).

### Risk of bias within studies

The risk of bias tables is included in S3 Table. The overall quality of the studies was poor for the 8 included trials (S1 and S2 Figs). Only 1 study had addressed all features of a randomised trial adequately, with low risk of biases.[26] Three areas of quality assessment were of particular concern. Firstly, there was inadequate randomisation. Two of the trials included a combination of randomised and pseudo-randomised design. In Engstrom (1984), 28/ 210 (13%) patients with prior heart disease were directly assigned to treatment arm of STZ/5FU, rather than excluded from the trial using predefined exclusion criteria in the first place. In Sun (2005), 73/249 (29%) of patients received direct assignment: patients with previous heart disease or prior doxorubicin (Dox) were directly assigned with STZ/5FU; patients with previous renal disease or prior STZ were directly assigned with Dox/5FU; and patients with prior doxorubicin and 5FU were directly assigned with DTIC (dacarbazine). The randomised arms in these 2 trials were separately available, so only these were included in the meta-analyses. There was also significant imbalance between baseline characteristics in one study (prior surgery, P = 0.036 between treatment arms),[24] and no baseline characteristics were provided at all in a small study of 20 patients.[18] The inadequate randomisation process may significantly increase the risk of selection bias. Secondly, there was lack of blinding. None of the trials reported the use of placebo, and since treatment schedules and route of administration were different between treatment arms, blinding of trial participant was absent in all trials. This could significantly affect the patient-reported outcome of toxicity. Another significant issue was the lack of blinding in outcome assessment. Response was assessed biochemically (5-HIAA) and radiologically/clinically, as opposed to the single now-standard RECIST criteria. None of these trials reported the use of central radiology, even though most of these trials were multi-centred clinical trials. Importantly, 4 of the 7 trials- all conducted in the 70s and 80s- allowed the use of clinical measurement of tumors and hepatomegaly, and considered these as measurable diseases.[20–23] Clinical measurement of tumors and liver spans using callipers are highly variable and clinician dependent. This, combined with the lack of blinding of assessors, has introduced very high risk of measurement bias into these randomised trials. Thirdly, there was incomplete outcome data. Only 2 trials used intention-to-treat analysis [25,26]. The loss of patients in outcome measurements were a particular issue in 3 trials, where 18% could not be accounted for survival,[20] and 18–54% were unaccounted for toxicity outcome measurement.[21,22] Other losses of data considered in the critical appraisal of these trials were the lack of reporting of statistical description/ calculations in 2 trials,[21,22] and the lack of long-term survival data beyond 1 year of follow up.[18] Selective reporting bias was suspected to be present.

### Measurement of effect

#### Response rate of STZ/5FU versus other chemotherapies and IFN

**Fig 2:**
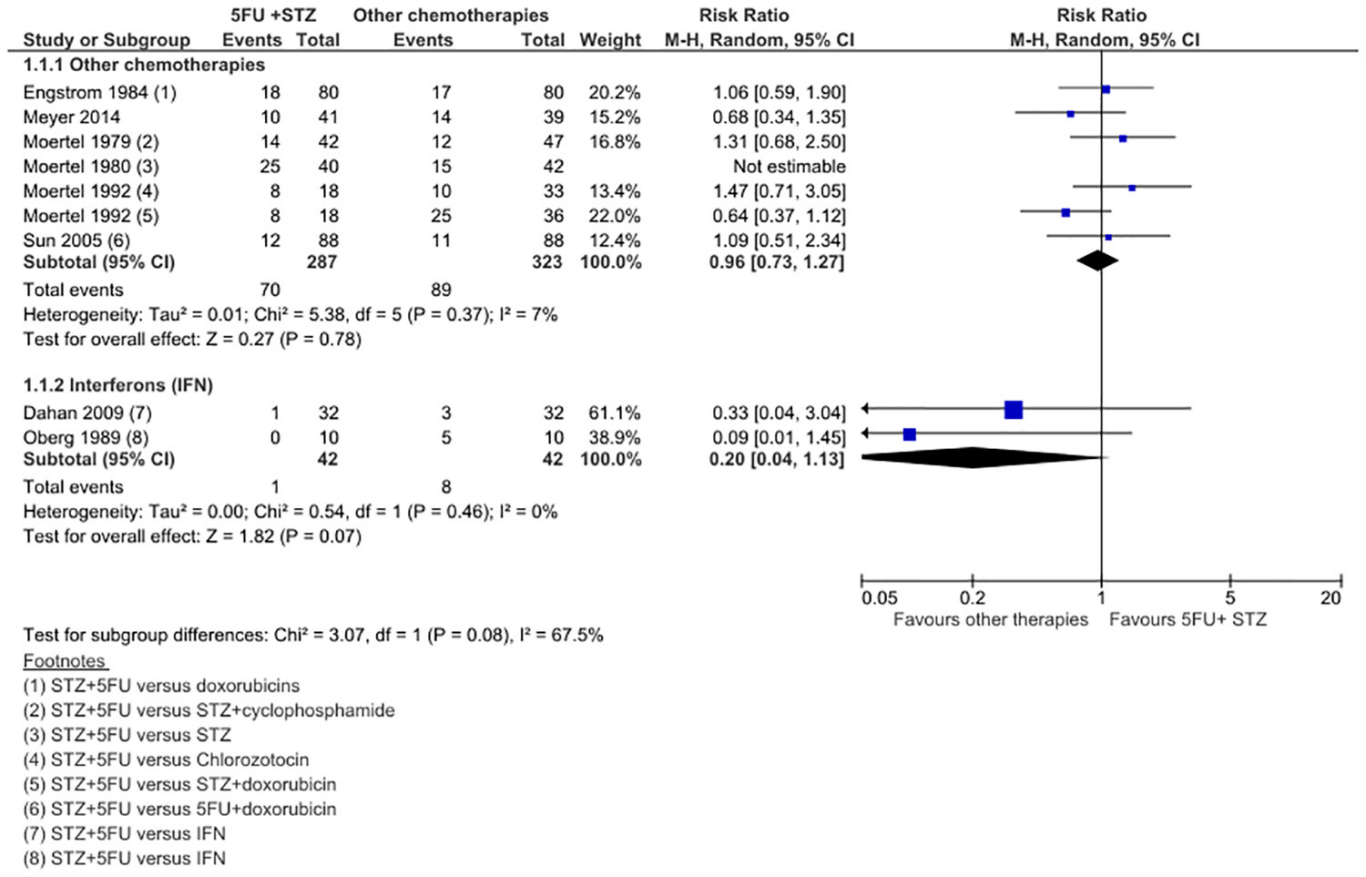
Forest plot of comparison 1: Response Rate STZ+5FU versus chemotherapy of IFN.

The primary objective was to meta-analyse only studies with low risk of bias. This could not be performed as only one study had a low risk of bias.[26] A sensitivity analysis combining all trials was performed, though only a conservative conclusion could be made in light of this. In 5 trials comprising of 610 patients, STZ/5FU was no different to other chemotherapies in inducing tumor response (RR 0.96; CI 0.72 to 1.27) (Fig 2). Moertel (1980) was an original trial that established the role of STZ/ 5FU. In this trial, STZ was the comparator arm and STZ/5FU were the experimental arm.[22] Adding Moertel (1980) did not make any significant changes to the results, only increasing the level of statistical heterogeneity further from 7% to 42% (I^2^). On a-priori subgroup analysis, Doxorubicin-based combination was studied in 2 trials in 248 patients. There was a suggestion that STZ/Dox and 5FU/Dox could be better than STZ/5FU (RR 0.79; CI 0.47 to 1.32), but this result was not significant. Other a-priori subgroup analyses based on the tumor subtypes and known prognostic factors could not be performed, as study data was not separately available for these variables. Whilst IFN appeared to induce a much better tumor response in 2 trials comprising of 84 patients (RR 0.20; CI 0.04 to 1.13), this result was not significant with a wide CI. No statistical heterogeneity was observed, but the number of events observed was small (9/84) and publication bias was likely present (S3 Fig). This estimate was likely far from the true effect and should be disregarded. Subgroup analyses could not be performed in this analysis, since there were only 2 trials included in this meta-analysis.

#### Progression Free Survival of STZ/5FU versus other chemotherapies and IFN

**Fig 3:**
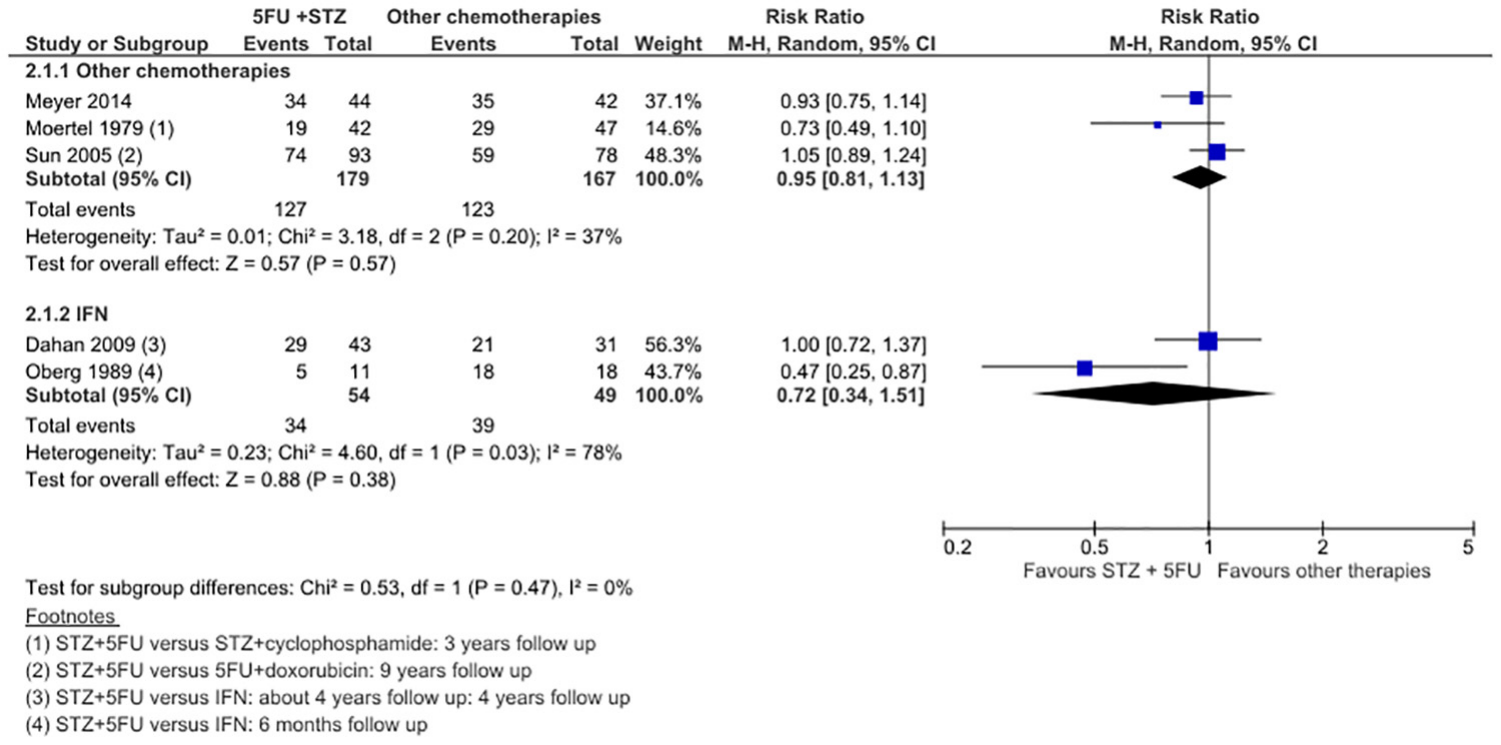
Forest plot of comparison 2: Progression-free survival upon completion of follow up, STZ+5FU versus chemotherapy or IFN.

5 studies were included (3 comparing against other chemotherapies, 2 against IFN) for the meta-analysis of PFS rate. In 3 trials comprising 346 patients, STZ/5FU was no different to other chemotherapies in improving PFS (RR 0.95, 95% CI 0.81 to 1.13). In 2 studies of 103 patients, STZ/5FU was no different to IFN in improving PFS (RR 0.72, 95% CI 0.34–1.51). (Fig 3) However, significant heterogeneity was present for both STZ/5FU versus chemotherapy (I^2^ = 37%, P = 0.20) and STZ/5FU versus IFN (I^2^ = 78%, P = 0.03). This may be due to variations in follow up time. All three studies with follow up times <4 years—Moertel (1979), Oberg (1989) and Meyer (41)—reported risk ratios favouring other therapies (RR 0.73, 0.47 and 0.93 respectively). In comparison, the two trials with longer follow up (4 years; 9 years) –Dahan (2009) and Sun (2005)—reported risk ratios closer to the null effect (RRs 1.00 and 1.05). Median PFS was not statistically different between treatment arms in all included studies (Table 1).

**Table 1:** Median Progression-free and Overall Survival.

Study	Intervention	Comparator	PFS- Intervention	PFS- Comparator	P-value	OS intervention	OS comparator	P value
**Engstrom 84**	Doxorubicin	STZ+5FU				12.0m	16.1m	P = 0.25
**Moertel 79**	STZ+Cyclophosphamide	STZ+5FU	7m	6.5m	Not significant (NS)	11.5m	6.8m	Not reported
**Moertel 80**	STZ+5FU	STZ	-	-	NS	26m	16.4m	NS
**Moertel 92**	STZ+Dox	STZ+5FU	21m	13m	Not reported	26.4m	16.8m	**P = 0.01**
**Moertel 92**	Chlorozotocin	STZ+5FU	22m	13m	Not reported	18m	16.8m	NS
**Sun 05**	STZ+Dox	STZ+5FU	4.5m	5.3m	P = 0.17	24.3m	15.7m	**P = 0.027**
**Dahan 09**	IFNα-2A	STZ+5FU	14.1m (6.7–21.2)	5.5m (2.9–25)	P = 0.25	44.3m	30.4m	P = 0.83
**Oberg 89**	IFNα-2A	STZ+5FU	-	-	N/A	-	-	N/A
**Meyer 14**	STZ+Capecitabine+Cisplatin	STZ+Capecitabine	9.7m	10.2mo	Not reported; HR 1.35(95% CI 0.83–2.17)*	9.7m	10.2m	Not reported; HR 0.86 (95% CI 0.48–1.54)*

*Figures derived from publication; given that the intervention and comparator arms were reported the other way around, the hazard ratio here was derived from the inverse of the published hazard ratio/95% CI.

#### Overall Survival of STZ/5FU versus other chemotherapies and IFN

**Fig 4:**
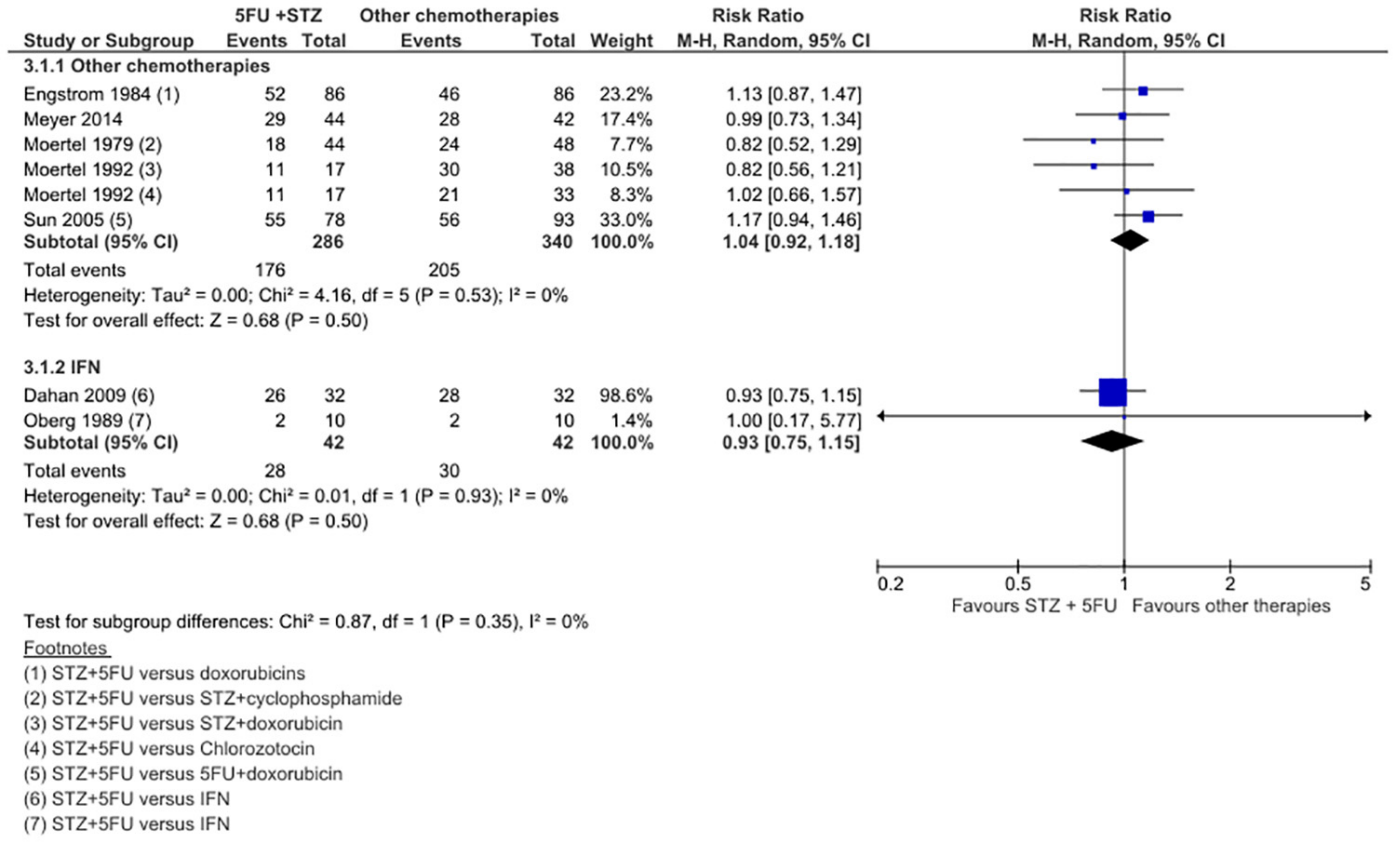
Forest plot of comparison 3: 1-Year Overall Survival, STZ+5FU versus chemotherapy or IFN.

**Fig 5:**
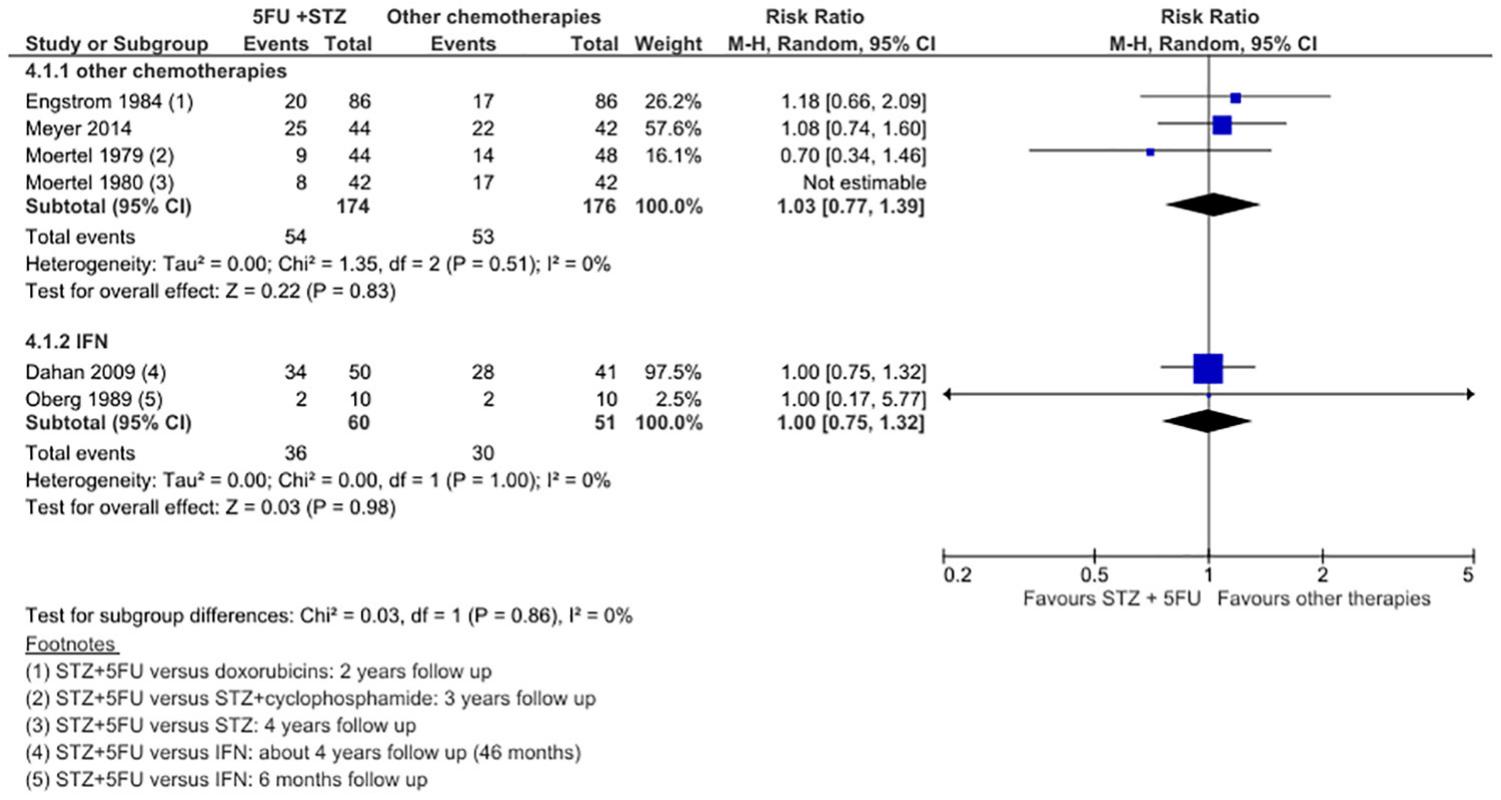
Forest plot of comparison 4: Overall Survival upon completion of follow up, STZ+5FU versus chemotherapy or IFN.

In 5 studies of 626 patients, STZ/5FU was no different to other chemotherapies in improving 1-year OS rate (RR 1.04; 95% CI 0.92 to 1.18) (Fig 4). In 2 studies of 84 patients, IFN also did not improve OS over STZ/5FU (RR 0.93; 95% CI 0.75 to 1.15). There was no significant statistical heterogeneity in either meta-analysis. No subgroup differences were observed between follow-up times [(<4 years: (RR 1.00; 95% CI 0.84 to 1.20); >4 years: (RR 0.98; 95% CI 0.76 to 1.26)], or in doxorubicin-based regimens (RR 1.02; 95% CI 0.72 to 1.43). OS upon completed follow up was only available for chemotherapy versus STZ/5FU comparison in 3 studies with 350 patients (RR 1.03; 95% CI 0.77 to 1.39) (Fig 5), and for IFN versus STZ/5FU comparison in 2 studies with 111 patients (RR 1.00; 95% CI 0.75 to 1.32). Adding Moertel (1980) did not change the estimated OS. Median OS was available for 8 included studies. Both Moertel (1992) and Sun (2005) demonstrated significantly increased OS with STZ/dox (26.4m v 16.8m, P = 0.01) and 5FU/dox (24.3m v 15.7, P = 0.027) compared to control (Table 1). It is important to note that 4 trials allowed cross-over after tumor progression,[20,21,23,24] so any beneficial effect attributed to the intervention/experimental arms was likely diluted.

#### Toxicities of STZ/5FU, other chemotherapies and IFN

Reporting measures for toxicity varied between trials, and only two studies used the standardised measurement of WHO or CTCAE criteria.[26] [25] [20, 22] Meta-analyses were performed for overall (or all grades) toxicity and severe (or WHO grade 3–4 equivalent) haematological and renal toxicity. In general, the rates of haematological toxicity were fairly high among chemotherapies (40–80%), though the severe toxicity rate only ranged from 5–33%. Renal toxicity rates were variable (2–46%) among trials, and severe toxicity was low (0–15%). Substantial heterogeneity was observed for nearly all comparisons (I^2^ = 28%-74%), possibly reflecting the large variations in the non-standard definitions of toxicities in each trial. From these meta-analyses, there was no difference between standard STZ/5FU and other chemotherapies in the rates of overall (RR 0.97; CI 0.75 to 1.25) or severe (RR 0.99; CI 0.49 to 1.99) haematological toxicities, and overall (RR 0.98; CI 0.59 to 1.63) or severe (RR 0.89; CI 0.33 to 2.43) renal toxicities. IFN appeared to cause significantly higher rates of overall haematological toxicity (RR 0.47; CI 0.27 to 0.82) and lower rates of renal toxicity (RR 3.61; CI 1.24 to 10.51), but the rate of severe toxicities were not significantly different in one study (S4–S7 Figs, S4 and S5 Tables).[26] Other important aspects of toxicity included nausea and vomiting, which was up to 85% in incidence among chemotherapies, though this was before the era of modern 5-HT3 and aprepitant anti-emetic. Flu-like symptoms were also higher in IFN (25%, 78%) than STZ/5FU (0%, 6%) in the two IFN trials.[18,26]

## Discussion

### Summary of evidence

The management of NETs depends on their stage, tumor bulk, functional status and grade. Complete surgical resection remains the only curative treatment option. Whilst chemotherapy continues to be recommended as an option in clinical guidelines for patients with pancreatic NET (PNETs), midgut NET and neuro-endocrine carcinoma (G3) of any site, these recommendations are largely based on weak evidence from non-randomised single arm retrospective studies to older randomised trials with questionable study designs.[3,27] In its original design, this systematic review aimed at meta-analysing high quality RCTs. However, only one trial was considered low risk of bias.[26] A sensitivity analysis including all trials regardless of risk of bias was performed nonetheless. We found no difference between STZ/5FU and other chemotherapies in response rate, PFS, 1-year OS and OS. Two studies reported significantly improved median OS of STZ/dox and 5FU/dox compared to STZ/5FU,[23,24] though median PFS was no different between chemotherapy regimens. All grades or severe (grade 3/4) toxicities were no different between any chemotherapy regimens. As for IFN, whilst response rates appeared to be higher, no PFS or OS were demonstrated. Significantly higher overall haematological and lower overall renal toxicities were associated with IFN, but the event rates of severe haematological and renal toxicity were extremely small and no different between STZ plus 5-FU and interferon.

### Limitations

There were three levels of limitations to this current systematic review. These were essentially related to the absence of strong evidence in the current literature (review level), low quality of the studies (study level) and weak outcome measures (outcome level). On the review level, a comprehensive search was performed on current literature, but only 257 articles were identified as potentially relevant, and >30 chemotherapy NET trials were excluded due to retrospective, non-randomised or non-controlled study designs. Publication bias was likely present for the IFN and chemotherapy comparative trials. No randomized studies of modern chemotherapy such as temozolomide were identified; non-randomized studies on this and new chemotherapeutic agents/combinations thereof could not be included in the current systematic review. On the study level, inadequate randomization, lack of blinding, missing data and selective reporting had resulted in selection, measurement, attrition and reporting biases, which adversely affected the quality of the included studies. Finally, this systematic review was limited by the weak outcome measures. It has been suggested that PFS or delay in progression should be used as study endpoints of neuroendocrine tumors given its variable and generally long survival. OS, on the other hand, is unreliable since most patients would have various forms of salvage surgery after tumor progression.[28] In this systematic review, the only PFS-related endpoints were median PFS and progression free rate. Median PFS could not be meta-analysed due to a lack of reporting of standard deviation. PFS rate was not comparable due to high statistical heterogeneity, and was strongly biased by the time to follow up. Comparing two trials with long versus another two with relatively short follow up (>4 years versus <4 years), the estimates for PFS rate were much closer to the null value for studies with longer follow up. For OS, median OS and OS rate had similar issues as median PFS and PFS rate. The estimated 1 year OS rate was also unreliable given the long survivals of patients with this cancer. Response rate assessment was also a major issue in most of these trials, given the lack of central assessment, and inclusion of clinical and biochemical assessment in the definition of tumor response. All included studies used urinary 5-HIAA as a surrogate marker of biochemical response, except for Dahan (2009) which utilised both urinary 5-HIAA and NSE. Urinary 5-HIAA is generally elevated in patients with metastatic midgut carcinoid tumors, but not useful in patients with foregut, hindgut or pancreatic NET.[28] Chromogrannin A (CGA) is somewhat specific (86%) and moderately sensitive (68%), but due to the lack of other superior biomarkers now considered one of the standard biomarkers in NET.[3,29,30] Most of these trials predated the introduction of CGA in routine practice, but the use of urinary 5-HIAA alone nevertheless introduce measurement bias by today's standards. Lastly, toxicity assessment was also widely heterogeneous among the included studies. Only 1 study used the standard CTCAE/ WHO criteria of grading of toxicity.[26] Other studies had different definition of toxicities, and thus none of this information could be integrated in meta-analysis.

The other difficulty in interpreting the available data was the increasing understanding of NET biology and changes in NET classification that have occurred particularly in the last 10 years. The WHO classification of 2010 has provided a way to classify NETs and direct treatment based on that classification. Cisplatin/etoposide (with potential substitution of carboplatin for cisplatin) is often used in G3 tumors as is the combination of temozolomide and capecitabine (CAPTEM) particularly for tumors with Ki67 <55% where platinum doublet chemotherapy is associated with a lesser response rate [31]. Whilst some prior studies classified response by subgroups of well-differentiated and poorly differentiated tumors (eg Moertel 1991), analysis by histological subgroups according to the WHO classification was not possible.

The evolution of an impressive array of systemic options over the last few years has also changed the place of chemotherapy in the treatment paradigm for NET. Multiple positive randomized trials for lanreotide (CLARINET) [32], everolimus—both in pancreatic primaries (RADIANT-3) [33] and more recently GI/lung primaries (RADIANT-4) [34], as well as the recent impressive results favouring PRRT over increased dose octreotide in midgut NETs (NETTER-1) have all provided potential dilemmas to the clinician regarding choice and sequencing of the above therapies. As welcome as this dilemma is, it remains to be seen whether chemotherapy shows enough efficacy compared to these options to warrant its side effect profile. In recent times, newer chemotherapy regimens with temozolomide, alone or in combination with thalidomide or capecitabine, have shown promising activity.[35–37] In a study with temozolomide and capecitabine (CAP-TEM), radiological response was reported to be as high as 70%, with an above-average PFS of 18 months and a tolerable side effect profile. Trials such as E2211 (comparing treatment with CAPTEM to temozolomide alone in PNETs) are greatly welcome in that they incorporate newer promising agents with response evaluation according to RECIST criteria.

## Conclusion

Although this systematic review has the above limitations, it remains the largest and most comprehensive review and appraisal of the existing evidence of chemotherapy in NET. The limitations of this systematic review were related to the limitations of the individual studies, so this review in fact highlights the paucity of strong evidence in this field as well as an urgent need of good randomised data in chemotherapy in NET. Whilst new chemotherapeutic agents show promise and are being trialled, no current randomized data exist regarding their use. Until such randomised study data becomes available, the existing evidence of chemotherapy in NET is not strong compared to well-designed large positive randomised phase III trials on somatostatin (PROMID), everolimus (RADIANT-2, RADIANT-3) and sunitinib, and caution must therefore be exercised in applying chemotherapy data on NET patients.

## Supporting Information

S1 Fig Risk of bias graph for included trials. (TIF)

S2 Fig Risk of bias summary for included studies. (TIF)

S3 Fig Funnel plot of comparison: 1 Response Rate, outcome: 1.1 STZ+5FU versus other chemotherapies and IFN. (TIF)

S4 Fig Forest plot of comparison 5: All grades haematological toxicity, STZ+5FU versus chemotherapy or IFN. (TIF)

S5 Fig Forest plot of comparison 6: Grade 3/4 haematological toxicity, STZ+5FU versus chemotherapy or IFN. (TIF)

S6 Fig Forest plot of comparison 7: All grades renal toxicity, STZ+5FU versus chemotherapy or IFN. (TIF)

S7 Fig Forest plot of comparison 8: Grade 3/4 renal toxicity, STZ+5FU versus chemotherapy or IFN. (TIF)

S1 PRISMA Checklist (DOC)

S1 Table MEDLINE/ EMBASE search strategy. (DOCX)

S2 Table Study characteristics. (DOCX)

S3 Table Risk of bias table. (DOCX)

S4 Table All grades toxicity, % Data for both direct assignment and randomised groups were included. # Flu-like symptoms occurred in 14/18 (78%) IFN and 0/11 (0%) STZ/5FU (Oberg 89). # Fever (any grade) occurred in 8/32 (25%) IFN and 2/32 (6%) STZ/5FU (Dahan 09).
(DOCX)

S5 Table Grade 3/4 toxicity, % Data for both direct assignment and randomised groups were included. ^ P-value for overall maximal toxicities.
(DOCX)
