# Supplementary figures and images for: Systematic Review and Meta-Analysis on the Role of Chemotherapy in Advanced and Metastatic Neuroendocrine Tumor (NET)
Source: PLoS One. 2016 Jun 30;11(6):e0158140. doi: 10.1371/journal.pone.0158140 (PMC4928873; doi:10.1371/journal.pone.0158140)

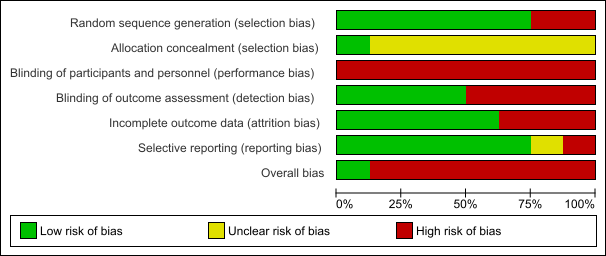

Supplement: S1 Fig — (TIF) [file pone.0158140.s001.tif]

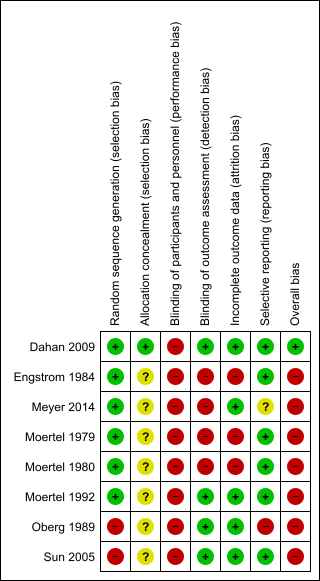

Supplement: S2 Fig — (TIF) [file pone.0158140.s002.tif]

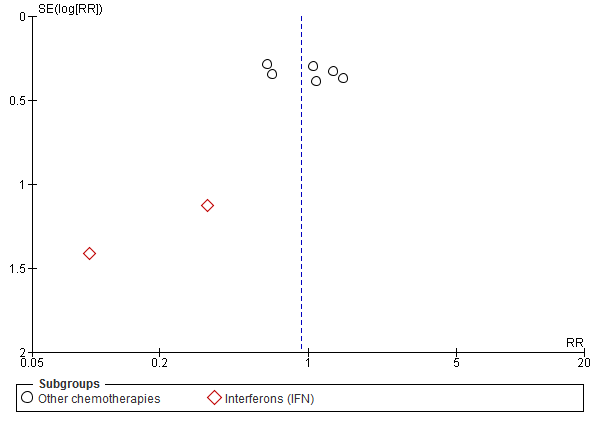

Supplement: S3 Fig — (TIF) [file pone.0158140.s003.tif]

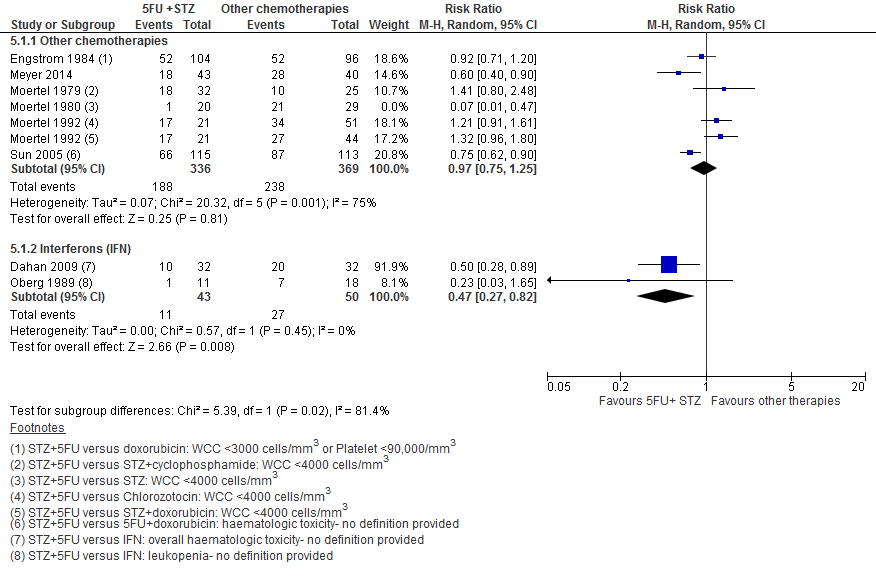

Supplement: S4 Fig — (TIF) [file pone.0158140.s004.tif]

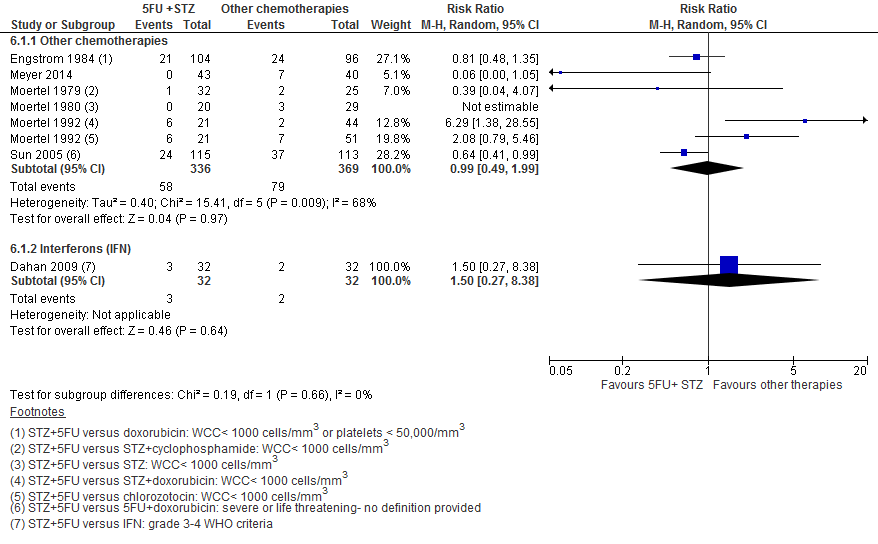

Supplement: S5 Fig — (TIF) [file pone.0158140.s005.tif]

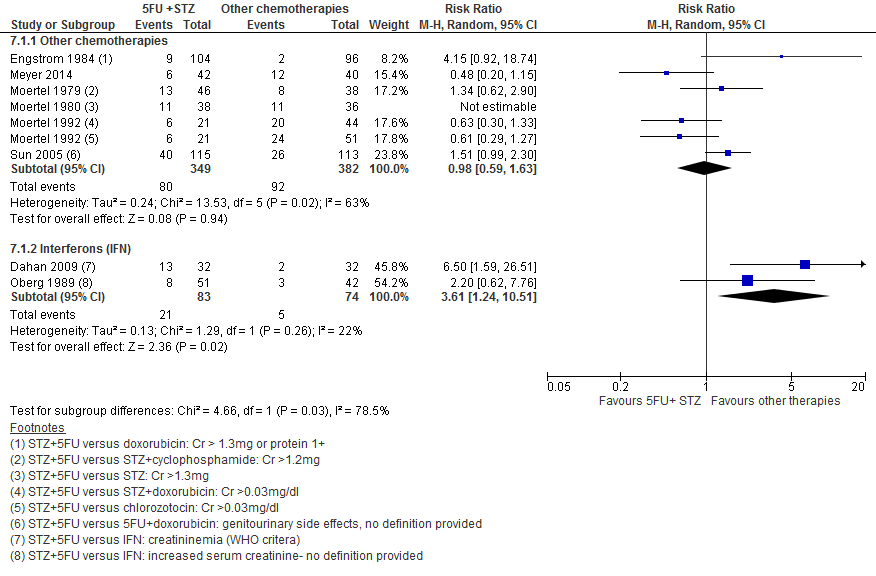

Supplement: S6 Fig — (TIF) [file pone.0158140.s006.tif]

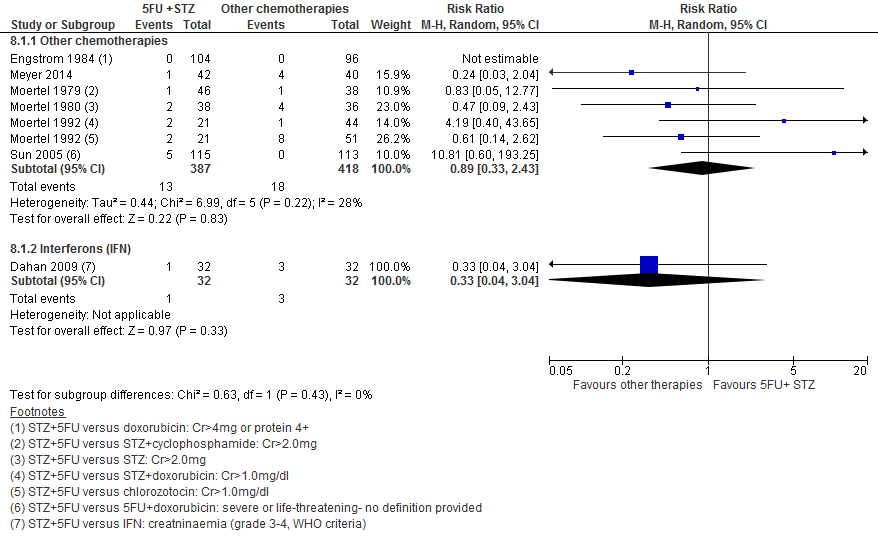

Supplement: S7 Fig — (TIF) [file pone.0158140.s007.tif]
