# Supplementary material for: Systematic Review and Meta-Analysis on the Role of Chemotherapy in Advanced and Metastatic Neuroendocrine Tumor (NET)
Source: PLoS One. 2016 Jun 30;11(6):e0158140. doi: 10.1371/journal.pone.0158140 (PMC4928873; doi:10.1371/journal.pone.0158140)
Supplement: S1 Table — (DOCX) [file pone.0158140.s009.docx]

Supplementary Table 1: MEDLINE/ EMBASE Search Strategy

1. Drug Therapy/

2. Antineoplastic Agents/

3. chemotherap$.tw.

4. Streptozocin/

5. Streptozocin$.tw.

6. Fluorouracil/

7. fluorouracil$.tw.

8. Doxorubicin/

9. Doxorubicin$.tw.

10. Dacarbazine/

11. Dacarabazine$.tw.

12. DTIC.tw.

13. temozolomide/

14. Temozolomide$.tw.

15. or/1-14

16. Neuroendocrine Tumors/

17. Carcinoid Tumor/

18. Insulinoma/

19. Gastrinoma/

20. neuroendocrine$.tw.

21. carcinoid$.tw.

22. insulinoma$.tw.

23. gastrinoma$.tw.

24. (islet cell adj10 tumo$).tw.

25. APUD.tw.

26. or/16-25

27. randomized controlled trial.pt.

28. controlled clinical trial.pt.

29. randomized.ab.

30. placebo.ab.

31. clinical trials as topic/

32. randomly.ab.

33. trial.ti.

34. or/27-33

35. Review.pt. and Medline.tw.

36. Meta analysis.pt.

37. (systematic$ and (review$ or overview$)).tw.

38. meta?analy$.tw.

39. meta analy$.tw.

40. or/35-39

41. 15 and 26 and 34

42. 15 and 26 and 40

43. limit 41 to ("all adult (19 plus years)" and humans and ("reviews (maximizes sensitivity)" or "therapy (maximizes sensitivity)"))
