# Supplementary material for: Systematic Review and Meta-Analysis on the Role of Chemotherapy in Advanced and Metastatic Neuroendocrine Tumor (NET)
Source: PLoS One. 2016 Jun 30;11(6):e0158140. doi: 10.1371/journal.pone.0158140 (PMC4928873; doi:10.1371/journal.pone.0158140)
Supplement: S2 Table — (DOCX) [file pone.0158140.s010.docx]

Supplementary Table 2: study characteristics

### Dahan 2009 / Seitz 2006

| **Methods** | Phase 3 Randomised controlled study. Multi-centred (23 centres in France). 1998-2004. n=64. |
| --- | --- |
| **Participants** | Unresectable malignant carcinoid tumour (well- differentiated endocrine carcinoma with positive Grimelius or Chromogrannin A staining) with documented tumour radiological or biochemical progression. WHO PS 0-2  **Patient characteristics:** Mean age 65 years, 13% WHO PS 2, 63% mid-gut, 99% with progressive and measurable disease, 84% with liver metastases |
| **Interventions** | Randomised 1:1 to Streptozotocin (STZ) and 5-FU (comparator) versus Interferon Alpha (intervention)  Arm A: 5FU (400mg/m^2^) + STZ (500mg/m^2^) D1-5 in a 6-week cycle  Arm B: IFN-a-2A 3x10^6^ 3x weekly in a 6-week cycle  Treatment was planned for 2 (min) to 8 (max) cycles. Patients with stable disease (SD) continued allocated therapy |
| **Outcomes** | **Primary endpoint**: 1-year PFS  **Secondary endpoints:** TTP, overall survival, toxicity/ tolerability  **Follow up:** Clinical exam every 6 weeks, CT and biochemistry (incl. 5-HIAA and NSE) every 12 weeks |
| **Notes** | Concomitant somatostatin analogues allowed (17%) Prior immunotherapy, radiotherapy to primary, or chemotherapy <6 weeks were excluded |

### Engstrom 1984

| **Methods** | Randomized phase 2/3 study plus direct assignment. Multi-centred. 1976-81. n=232 |
| --- | --- |
| **Participants** | Progressive Metastatic NET confirmed by histology. ECOG 0-3, measurable tumour mass or malignant hepatomegaly.  **Patient characteristics:** median age 60 years, 26% ECOG PS2-3, only 36% mid-gut (but 44% unknown), |
| **Interventions** | Randomised 1:1 STZ and 5-FU versus Doxorubicin. Direct treatment 5FU and STZ for patients with heart disease  Arm A: 5-FU (400 mg/m^2^) D1-5, D36-40 +STZ (500 mg/m^2^) D1-5 10-week cycle  Arm B: Doxo (60 mg/m^2^) on d1, 22, 43 and every 4 weeks.  Crossover allowed in case of progressive disease.172 patients randomised. Rest 38 with heart disease or prior chemo received direct therapy. |
| **Outcomes** | **Endpoints:** Surivlval, response, toxicity  **Follow up:** every 10 weeks, CT and biochemistry (5-HIAA) |
| **Notes** | Therapy deferred after major surgery, radiation or chemotherapy for 4 weeks  Randomised data were separately available, and these data was therefore included. |

### Moertel 1979

| **Methods** | Randomised controlled trial. Multi-centred (27 centres). n=118 |
| --- | --- |
| **Participants** | Unresectable metastatic carcinoid tumour with measurable disease ("clinically" by ruler or caliper, or 5HIAA). ECOG 0-3  **Patient characteristics:** median age 60 years, 31% ECOG 2-3, 51% midgut. Prior chemotherapy 14% only. |
| **Interventions** | Randomised 1:1 STZ and 5FU versus STZ and Cyclophosphamide  Arm A: 5-FU (400 mg/m^2^) + STZ (500 mg/m^2^) D1-5 6-week cycle  Arm B: Cyclophosphamide (1000 mg/m^2^) D1 3-week cycle + STZ (500 mg/m^2^) D1-5 6-week cycle  Cross-over allowed after progression |
| **Outcomes** | **Endpoints:** response, survival, toxicity  **Follow up:** every 6 weeks clinical examination, tumour measurement (calipers or ruler) and 5HIAA |
| **Notes** | Therapy deferred after major surgery, radiation or chemotherapy for 4 weeks |

### Moertel 1980

| **Methods** | Randomised controlled trial. Multi-centred (28 centres). 1972-1978. n=84 |
| --- | --- |
| **Participants** | Unresectable advanced Islet cell Carcinoma, confirmed by histology. ECOG 0-4. Measurable malignant disease (clinically measurable tumour, malignant hepatomegaly, biochmical or radiological)  **Patient characteristics:** median age 53 years, 24% ECOG 2-4, 51% with functioning tumours, no information on location of tumour |
| **Interventions** | Randomised 1:1 STZ versus STZ and 5FU  Arm A: STZ (500 mg/m^2^) D1-5 6-week cycle  Arm B: STZ (500 mg/m^2^) + 5FU (400 mg/m^2^) D1-5 6-week cycle |
| **Outcomes** | **Endpoints:** response, survival, toxicity  **Follow up:** every 6 weeks evaluation of therapeutic response, which might include clinical, laboratory and/or radiological assessment |
| **Notes** | Therapy deferred after major surgery (2-3 weeks), radiation (4 weeks) or chemotherapy (4 weeks) |

### Moertel 1992

| **Methods** | Randomised controlled trial. Multi-centred. 1978-1985. n=125 |
| --- | --- |
| **Participants** | Advanced Islet cell Carcinoma, confirmed by histology. ECOG 0-3. Measurable malignant disease (clinically measurable tumour, malignant hepatomegaly, laboratory or radiological)  **Patient characteristics:** median age 54 years, 30% ECOG 2-3, 48% with documented functioning tumour, no information on location of tumour |
| **Interventions** | STZ and 5FU versus STZ and doxorubicin versus Chlorozotocin  Arm A: STZ (500 mg/m^2^) + 5FU (400 mg/m^2^) D1-5 6-week cycle  Arm B: STZ (500 mg/m^2^) + 5FU (400 mg/m^2^) D1, 22 6-week cycle  Arm C: Chlorozotocin (150 mg/m^2^) 7-week cycle  Cross-over upon tumour progression. If progressed on arm C, patients were randomised to Arm A or B. If progressed on Arm A or B, patients proceeded to Arm C. (n=31) |
| **Outcomes** | **Endpoints:** response, survival, toxicity  **Follow up:** every 6-7 weeks evaluation of therapeutic response, which might include clinical, laboratory and/or radiological assessment |
| **Notes** | Therapy deferreed after major surgery (3 weeks), radiation (4 weeks) or chemotherapy (4 weeks) |

### Oberg 1989

| **Methods** | Randomised controlled trials. Single Instituition. n=20 |
| --- | --- |
| **Participants** | Metastatic carcinoid tumours (liver metastases) with active carcinoid symptoms  **Patient characteristics:** Mean age 62, no information on PS. 95% Midgut (Ileum primary). All had carcinoid symptoms (6/20 had heart failure).  However, no description of patient characteristics between treatment groups. |
| **Interventions** | STZ and 5FU versus IFN (Cantell type PIF A and B)  Arm A: STZ (1000mg) + 5FU (400 mg/m^2^) D1-3 6-weekly cycle  Arm B: IFN 6MU daily D1-3 6-weekly cycle  No other treatment for carcinoid syndrome was used |
| **Outcomes** | **Endpoints:** response, survival, toxicity  **Follow up:** CT/ USS every 3rd month, Laboratory assessment |

### Sun 2005

| **Methods** | Randomised controlled trials plus direct assignment. Multi-centred (32 centres). 1981-1990. n=249 |
| --- | --- |
| **Participants** | Unresectable histologically proven carcinoid tumours. Measurable tumour by radiological or biochemical measures  **Baseline characteristics:** Median age 60, ECOG 2/3 22%, 31% Midgut, 41% with active carcinoid symptoms, 57% with prior surgery |
| **Interventions** | A combination of randomised study (5FU and doxorubicin versus 5FU and STZ), and non-randomised study (patients with previous heart disease or previous doxorubicin were directly assigned with 5FU and STZ; patients with previous renal disease or previous STZ were directly assigned with Doxorubicin and 5FU; patients with previous doxorubicin and 5FU were directly assigned with DTIC)  Arm A: 5-FU (400 mg/m^2^) D1-5, D36-40 +STZ (500 mg/m^2^) D1-5 10-weekly cycle  Arm B: Doxo (40 mg/m^2^) D1 + 5FU (400 mg/m^2^) D1-5 5-weekly cycle  Cross-over to DTIC after progression on either randomised arms was allowed |
| **Outcomes** | **Endpoints:** Response rate, PFS, Overall survival, Toxicity. The study was powered to detect significant difference in response rate, and overall survival (though author reported a calculation error of sample size for overall survival, with no provision for interim analyses)  **Follow up:** Patients were re-evaluated every 4 (DTIC)- 5 weeks (combination). It was assumed from description of baseline assessment that follow up assessment included radiological and biochemical (5-HIAA) measures |
| **Notes** | Cross over design on progression  Independent results for randomised study part were available, and this part of the study was therefore included. |

### Meyer 2014

| **Methods** | Randomised controlled trial. Multi-centred (13 centres). 2006-2010. n=83 |
| --- | --- |
| **Participants** | Chemonaive, histologically confirmed, unresectable, advanced and/or metastatic NETs of pancreas, other gastrointestinal foregut, or unknown primary site, ECOG<=2  **Baseline characteristics:** Median age 58, 48% midgut, 20% gastroduodenal, 32% unknown primary. |
| **Interventions** | Arm A: Capecitabine (625mg/m^2^) D1-21, STZ (1g/m^2^) D1 3-weekly cycle * 6  Arm A: Capecitabine (625mg/m^2^) D1-21, STZ (1g/m^2^) D1, cisplatin (70mg/m^2^) 3-weekly cycle * 6 |
| **Outcomes** | **Endpoints:** Response rate by RECIST, biochemical response, safety, PFS, Overall survival, QoL. Study was powered to aim for a response rate>60% but not for comparisons between either arm.  **Follow up:** Patients were assessed for adverse events every cycle, and followed for disease progression and survival every 12 weeks. Tumour assessment with CT scans – baseline, every 3 cycles on treatment, every 12 weeks until progression. |
| **Notes** |  |

### Meyer 2014

| **Methods** | Randomised controlled trial. Multi-centred (13 centres). 2006-2010. n=86 |
| --- | --- |
| **Participants** | Unresectable histologically proven NET of pancreas, gastrointestinal foregut, or unknown primary site suggestive of abdominal foregut origin. No prior chemotherapy. Measurable tumour by RECIST (version 1.0). ECOG PS<=2.  **Baseline characteristics:** Median age 58, 58% Male, 48% pancreatic, 36% with active carcinoid symptoms. |
| **Interventions** | Randomized study 1:1 to the below arms:  Arm A: Capecitabine 625mg/m2 po bd D1-21 + STZ 1g/m2 (2h infusion) D1 3-weekly cycle – for six cycles. Ongoing treatment allowed if evidence of benefit.  Arm B: Capecitabine 625mg/m2 po bd D1-21 + STZ 1g/m2 (2h infusion) D1 + Cisplatin 70mg/m2 (2h infusion) D1 3-weekly cycle. Ongoing treatment allowed if evidence of benefit. |
| **Outcomes** | **Endpoints:** Response rate (by RECIST), biochemical response, safety, PFS, Overall survival, QoL. The trial was not powered for comparisons between two arms.  **Follow up:** Patients were assessed for adverse events every cycle, and followed for disease progression and survival every 12 weeks. CT scans at baseline, every 3 cycles on treatment, and every 12 weeks until progression. re-evaluated every 4 (DTIC)- 5 weeks (combination). It was assumed from description of baseline assessment that follow up assessment included radiological and biochemical (5-HIAA) measures |
| **Notes** |  |
