# Supplementary material for: Systematic Review and Meta-Analysis on the Role of Chemotherapy in Advanced and Metastatic Neuroendocrine Tumor (NET)
Source: PLoS One. 2016 Jun 30;11(6):e0158140. doi: 10.1371/journal.pone.0158140 (PMC4928873; doi:10.1371/journal.pone.0158140)
Supplement: S3 Table — (DOCX) [file pone.0158140.s011.docx]

Supplementary Table 3: Risk of Bias Tables

### Dahan 2009 / Seitz 2006

| **Bias** | **Support for judgement** |
| --- | --- |
| Random sequence generation (selection bias) | **Low Risk:** Randomised with a minimisation process. Baseline characteristics similar |
| Allocation concealment (selection bias) | **Low Risk:** Central randomisation (by phone or fax) |
| Blinding of participants and personnel (performance bias) | **High Risk:** No blinding. Different administration route (IV for chemo, SC for interferon). No placebo |
| Blinding of outcome assessment (detection bias) | **Mod risk:** Radiological and biochemical analysis only. No mentioning of central independent radiological reviews among the 23 centres. Standardised WHO criteria used for radiological assessment (>=50%) and laboratory assessment (>=50%) |
| Incomplete outcome data (attrition bias) | **Low Risk:** ITT analysis. All patients accounted for. |
| Selective reporting (reporting bias) | **Low Risk:** All endpoints reported. Statistical calculation included. Of the pre-planned 120 patients, 64 patients were included (trial closed early) |
| Overall bias | **Low risk of bias:** possible detection bias from lack of central radiological assessment, which may affect outcome measures of response, PFS but not OS |

### Engstrom 1984

| **Bias** | **Support for judgement** |
| --- | --- |
| Random sequence generation (selection bias) | Of the 210 patients in the trial, only 172 patients without heart disease were randomised. 28 patients with prior heart history were directly assigned to 5FU plus STZ (13%). **High risk** if considering the study as a whole, but **low risk** if considering just the randomised part of the study, since baseline characteristics of the *randomised patient groups* are similar. |
| Allocation concealment (selection bias) | **Unclear Risk:** No information on allocation concealment |
| Blinding of participants and personnel (performance bias) | **High Risk:** No blinding. Different administration route (IV for chemo, SC for interferon). No placebo |
| Blinding of outcome assessment (detection bias) | **High Risk:** Radiological assessment not specifically mentioned. Malignant hepatomegaly was considered "measurable", in which case measurement was taken to be the liver span below the costal margin and xiphoid process. Measurement of liver span as an indicator was highly subjected to measurement bias. No mentioning of central independent radiological reviews. Standardised WHO criteria used for radiological assessment (>=50%) and laboratory assessment (>=50%) |
| Incomplete outcome data (attrition bias) | **High Risk:** Not all patients randomised were accounted for. 232 patients were entered in the study. 210 patients from randomised and direct assignemnt arms received treatment (reasons for exclusion included 7 cancelled by physicians, 1 with no follow-up data, 1 improperly entered, 11 ineligible, 2 with major modifications). 200 were evaluated for toxicity, 195 for tumour response, and 172 patients for survival, but no reasons were given for these large discrepancies, nor was the number broken down to numbers in randomised and non-randomised arms. |
| Selective reporting (reporting bias) | **Mod Risk:** Endpoints were not specified in methods, standard endpoints including overall survival, response and toxicity were included. Progression not included. Statistical methods briefly described, with sufficient cases to detect 20% difference with 80% power and 5% alpha. |
| Overall bias | **High Risk of bias:** randomised data for patients without heart disease were independently available, thus allowing this study to be included and randomised data meta-analysed without compromising quality. However, the lack of blinding and ITT analysis, and presence of selective reporting, have increased the risk of bias of this trial. |

### Moertel 1979

| **Bias** | **Support for judgement** |
| --- | --- |
| Random sequence generation (selection bias) | **Low Risk:** Patients were randomised to one of two treatment arms. Baseline characteristics similar between arms. |
| Allocation concealment (selection bias) | **Unclear Risk:** No information on allocation concealment |
| Blinding of participants and personnel (performance bias) | **High Risk:** Chemotherapy schedule different between arms. No placebo given |
| Blinding of outcome assessment (detection bias) | **High Risk.** All cases were evaluated clinically (measurable tumour or palpable hepatomegaly), with measurement by calipers/ rulers. These would clearly be measured by different clinicians in different centres- subject to huge measurement bias. Objective response = 50% or more reduction of product of largest perpendicular diameters or 50% or more reduction of urinary 5 HIAA |
| Incomplete outcome data (attrition bias) | **High Risk.** Of the 118 patients randomised, 89 patients were fully evaluable. Authors made a reasonable attempt accounting for these attrition, including 2 totally disabled patients, 5 with gross protocol violation, 3 voluntarily left the study, 3 with treatment records lost, and 14 with no baseline measurement. Toxicity assessment was available for 93 patients for nausea and vomiting, 54 and 84 patients for haematological and renal toxicity profiles, and no reasons were given for these. |
| Selective reporting (reporting bias) | **Low Risk:** Endpoints were not specified in methods, standard endpoints including overall survival, progression, response and toxicity were included. No sections on statistical calculation / analysis included. |
| Overall bias | **High Risk of bias:** the absence of radiological assessment and reliance of rulers/ calipers for clinical mesurement of tumour were subject to vast inter- and intra-observer measurement (detection) bias. Although reasonable attempt was made |

### Moertel 1980

| **Bias** | **Support for judgement** |
| --- | --- |
| Random sequence generation (selection bias) | **Low Risk:** Randomised allocation. Baseline characteristics similar between arms. |
| Allocation concealment (selection bias) | **Unclear Risk:** No information on allocation concealment |
| Blinding of participants and personnel (performance bias) | **High Risk:** No placebo given. One treatment arm utilised one chemotherapy, and another utilised two chemotherapy. Tumour response classified by radiological (50%- WHO) or 5-HIAA (50%) response. |
| Blinding of outcome assessment (detection bias) | **High Risk:** Both radiological and clinical measurement of tumours allowed, but it was not made clear the number of patients undergoing radiological as opposed to clinical (calipers, rulers) measurement or tumour or liver span. No mentioning of central independent radiological reviews. Tumour response classified by radiological (50%- WHO) or 5-HIAA (50%) response. |
| Incomplete outcome data (attrition bias) | **High Risk:** Of the 103 patients randomised, 84 were evaluated. Authors made reasonable attempts to account for losses for analysis in a table. Most patients not evaluated were ineligible (16), and 3 were cancelled. However, toxicity assessment was available for 74 (72%) and 49 (48%) patients for haematological and renal toxicity profiles, and no reasons were given for these. |
| Selective reporting (reporting bias) | **Low Risk:** Endpoints were not specified in methods, standard endpoints including overall survival, response and toxicity were included. Progression not reported. No sections on statistical calculation / analysis included. |
| Overall bias | **High Risk of bias:** probable detection bias from allowing clinical measurement of tumours instead of radiological assessment alone (though it was unclear how many patients actually had clinically rather than radiologically measurable tumours) |

### Moertel 1992

| **Bias** | **Support for judgement** |
| --- | --- |
| Random sequence generation (selection bias) | **Low Risk:** Randomly assigned after stratifying ECOG and indicator of response |
| Allocation concealment (selection bias) | **Unclear Risk:** No information on allocation concealment |
| Blinding of participants and personnel (performance bias) | **High Risk:** No placebo given. Different chemotherapy scheduling |
| Blinding of outcome assessment (detection bias) | **High Risk:** Assessment by clinical examination was again allowed in addition to chest films, radioisotope scans or CT scanning. Again centralised radiology was not mentioned. Tumour response classified by radiological (50%- WHO) or 5-HIAA (50%) response. |
| Incomplete outcome data (attrition bias) | **Low Risk:** Of 125 randomised patients, up-to-date information was available for 105. The reasons for not including the other 20 were ineligibility (18) and patient withdrawal (2). Hence, there was a low risk of attrition bias |
| Selective reporting (reporting bias) | **Low Risk:** Endpoints were not specified in methods; standard endpoints including overall survival, progression, response and toxicity were included. Standard description for statistical analysis was given, but no calculation of planned sample size was included |
| Overall bias | **High risk of bias:** probable detection bias from allowing clinical measurement of tumours instead of radiological assessment alone (though it was unclear how many patients actually had clinically rather than radiologically measurable tumours) |

### Oberg 1989

| **Bias** | **Support for judgement** |
| --- | --- |
| Random sequence generation (selection bias) | **Very High Risk:** Randomly assigned after stratifying for urinary 5-HIAA (< or >500 umol/24hr), age and sex. However, no baseline characteristics between treatment arms described, which was especially an important feature to assess randomisation in small trials |
| Allocation concealment (selection bias) | **Unclear Risk:** No information on allocation concealment |
| Blinding of participants and personnel (performance bias) | **High Risk:** No placebo given. Different route of administration |
| Blinding of outcome assessment (detection bias) | **Mod Risk:** Radiological assessment with both CT and USS. No central radiology assessment. Tumour response classified by radiological (50%- WHO) or 5-HIAA (50%) response. |
| Incomplete outcome data (attrition bias) | **Low Risk:** Data from the 20 randomised patients were all available. |
| Selective reporting (reporting bias) | **High Risk:** Tumour response and toxicity were reported. Survival not reported. Baseline characteristics not reported. Statistical analysis planned for 30 patients, but only 20 were included due to early stopping rule |
| Overall bias | **High risk of bias:** aside from the lack of overall survival reporting, the randomisation process was in question, given that sample size was only 20 and no baseline characteristics between treatment arms were reported. This was a crucial piece of information especially in a small trial, to assess if randomisation was complete |

### Sun 2005

| **Bias** | **Support for judgement** |
| --- | --- |
| Random sequence generation (selection bias) | **High Risk:** Of the 249 patients in study, 176 were included in the randomistion part. However, 73 patients (29%) were in the direct assignment part which had a prospective non-randomised design. Baseline characteristics were evaluated by Fisher's exact test for balance. Of the 11 covariates, 10 were well matched in the randomised patient groups, but proportion of patients with prior surgery were significantly different between the randomised arms (P=0.036). Patients were stratified by ECOG, response indicators, prior chemotherapy agents, active heart disease and active renal disease |
| Allocation concealment (selection bias) | **Unclear Risk:** No information on allocation concealment |
| Blinding of participants and personnel (performance bias) | **High Risk:** No placebo given. Different chemotherapy scheduling |
| Blinding of outcome assessment (detection bias) | **Mod Risk:** No mentioning of central independent radiological reviews. Standardised WHO criteria used for radiological assessment (>=50%). Response was defined by radiological assessment only, not biological assessment* |
| Incomplete outcome data (attrition bias) | **Low Risk:** Of the 176 patients in the non-randomised arm, 163 were analysable. Of the 13 randomised, 4 were cancelled before treatment (no reason given) and 9 were ineligible. There was some risk of attrition for the 4 patients cancelled after randomisation, thought the proportion for this was quite small (2%) |
| Selective reporting (reporting bias) | **Low Risk:** All endpoints reported. Though the planned sample size was not reported, the alpha, beta, and proportional effects were all reported to allow for a rough estimate of sample size (roughly 162) |
| Overall bias | **High risk of bias:** if only the randomised part was considered and included for meta-analysis, then there was only some risk of selection bias by the randomisation process owing to the slight imbalance of prior surgery between the two treatment arms. There was possible detection bias from likely lack of central radiological assessment, which could possibly affect outcome measures of response, PFS but not OS |

### Meyer 2014

| **Bias** | **Support for judgement** |
| --- | --- |
| Random sequence generation (selection bias) | **Low Risk: “Stratified random block method”** |
| Allocation concealment (selection bias) | **Unclear Risk:** No information on allocation concealment |
| Blinding of participants and personnel (performance bias) | **High Risk:** No placebo given. Different chemotherapy scheduling |
| Blinding of outcome assessment (detection bias) | **High Risk:** Retrospective central review of objective tumour response for 10% of randomly selected patients completing at least 3 cycles – but no results from this in manuscript. |
| Incomplete outcome data (attrition bias) | **Low Risk:** All 83 patients evaluable for safety. 2/40 in Experimental and 2/43 in Control arm did not undergo response assessment (5% in all). |
| Selective reporting (reporting bias) | **Mod Risk:** All above endpoints reported, but results of retrospective central review of radiology not reported |
| Overall bias | **High risk of bias:** Given that the retrospective central review of tumour response (the primary endpoint) was not reported in an open-label trial, response rate and radiological PFS have a high risk of being biased (although OS would not be affected). |
