# Supplementary material for: Systematic Review and Meta-Analysis on the Role of Chemotherapy in Advanced and Metastatic Neuroendocrine Tumor (NET)
Source: PLoS One. 2016 Jun 30;11(6):e0158140. doi: 10.1371/journal.pone.0158140 (PMC4928873; doi:10.1371/journal.pone.0158140)
Supplement: S4 Table — # Flu-like symptoms occurred in 14/18 (78%) IFN and 0/11 (0%) STZ/5FU (Oberg 89). # Fever (any grade) occurred in 8/32 (25%) IFN and 2/32 (6%) STZ/5FU (Dahan 09). (DOCX) [file pone.0158140.s012.docx]

**Supplementary Table 4: Summary of toxicity**

| **Study** | **Intervention (I)** | **Comparator (C)** | **Haematological (I vs. C)** | **P-value** | **Renal Toxicity (I vs. C)** | **P-value** | **Comments/ Definitions of toxicities endpoints** |
| --- | --- | --- | --- | --- | --- | --- | --- |
| **Engstrom 84** | Doxorubicin | STZ+5FU | 52/96 (54%) v. 52/104 (50%) | Not reported | 2/96 (2%) v. 9/104 (9%) | - | ^%^WCC<3,000 cells/mm3 or Plt <90,000/mm3; Cr> 1.3mg or protein 1+ |
| **Moertel 79** | STZ+Cyclophosphamide | STZ+5FU | 10/25 (40%) v. 18/32 (56%) | Not reported | 8/38 (21%) v. 13/46 (28%) | Not reported | WCC<4,000 cells/mm^3^; Cr> 1.2mg |
| **Moertel 80** | STZ+5FU | STZ | 21/29 (72%) v. 1/20 (5%) | Not reported | 11/36 (31%) v. 11/38 (29%) | Not reported | WCC<4,000 cells/mm^3^; Cr> 1.3mg |
| **Moertel 92** | STZ+Dox | STZ+5FU | 27/44 (62%) v. 34/42 (81%) | Not reported | 20/44 (46%) v. 12/42 (29%) | Not reported | WCC<4,000 cells/mm^3^; Cr> 0.03mg/dl |
| **Moertel 92** | Chlorozotocin | STZ+5FU | 34/51 (67%) v. 34/42 (81%) | Not reported | 8/51 (15%) v. 12/42 (29%) | Not reported | WCC<4,000 cells/mm^3^; Cr> 0.03mg/dl |
| **Sun 05** | STZ+Dox | STZ+5FU | 87/113 (77%) v. 66/115 (57%) | Not reported | 26/113 (23%) v. 40/115 (35%) | Not reported | ^%^"Severe" or "life-threatening": no definition for these terms were provided. |
| **Dahan 09^#^** | IFNα-2A | STZ+5FU | 10/32 (31%) v. 20/32 (63%) | P=0.003 | 6/32 (19%) v. 2/32 (6%) | NS | "Overall haematological toxicities", "creatininemia" (WHO criteria) |
| **Oberg 89^#^** | IFN | STZ+5FU | 7/18 (39%) v. 1/11 (9%) | Not reported | 3/42 (7%) v. 8/51 (16%) | Not reported | "Leukopenia"; "Increased serum creatinine": no definition for these terms were provided. |
| **Meyer 14** | STZ+Capecitabine | STZ+Capecitabine+Cisplatin | No composite; anaemia 6/39 (15%) vs 7/43 (16%), neutropaenia 7/40 (18%) vs 3/43 (7%) | Not reported | 12/40 (30%) vs 6/42 (14%) | Not reported |  |

***Table S4****: All grades toxicity,* ***%*** *Data for both direct assignment and randomised groups were included*

***#*** *Flu-like symptoms occurred in 14/18 (78%) IFN and 0/11 (0%) STZ/5FU (Oberg 89)*

***#*** *Fever (any grade) occurred in 8/32 (25%) IFN and 2/32 (6%) STZ/5FU (Dahan 09)*
