# Supplementary material for: Systematic Review and Meta-Analysis on the Role of Chemotherapy in Advanced and Metastatic Neuroendocrine Tumor (NET)
Source: PLoS One. 2016 Jun 30;11(6):e0158140. doi: 10.1371/journal.pone.0158140 (PMC4928873; doi:10.1371/journal.pone.0158140)
Supplement: S5 Table — ^ P-value for overall maximal toxicities. (DOCX) [file pone.0158140.s013.docx]

**Supp Tab 5: Summary of Grade 3/4 toxicity**

| **Study** | **Intervention (I)** | **Comparator (C)** | **Haematological (I vs. C)** | **P-value** | **Renal Toxicity (I vs. C)** | **P-value** | **Comments/ Definitions of toxicities endpoints** |
| --- | --- | --- | --- | --- | --- | --- | --- |
| **Engstrom 84** | Doxorubicin | STZ+5FU | 24/96 (25%) v. 21/104 (20%) | Not reported | 0/96 (0%) v. 0/104 (0%) | - | ^%^WCC<1,000 cells/mm3 or Plt <50,000/mm3; Cr> 4.0mg or protein 4+ |
| **Moertel 79** | STZ+Cyclophosphamide | STZ+5FU | 2/25 (8%) v. 1/32 (3%) | Not reported | 1/38 (3%) v. 1/46 (2%) | Not reported | WCC<1,000 cells/mm^3^; Cr> 2.0mg |
| **Moertel 80** | STZ+5FU | STZ | 3/29 (10%) v. 0/20 (0%) | Not reported | 4/36 (11%) v. 2/38 (5%) | Not reported | WCC<1,000 cells/mm^3^; Cr> 2.0mg |
| **Moertel 92** | STZ+Dox | STZ+5FU | 2/44 (5%) v. 11/42 (25%) | Not reported | 9/44 (2%) v. 3/42 (7%) | Not reported | WCC<1,000 cells/mm^3^; Cr> 1mg/dl |
| **Moertel 92** | Chlorozotocin | STZ+5FU | 7/51 (14%) v. 11/42 (25%) | Not reported | 7/44 (15%) v. 3/42 (7%) | Not reported | WCC<1,000 cells/mm^3^; Cr> 1mg/dl |
| **Sun 05** | STZ+Dox | STZ+5FU | 37/113 (33%) v. 24/115 (21%) | P=0.078^ | 0/113 (0%) v. 5/115 (4%) | P=0.078^ | ^%^"Severe" or "life-threatening": no definition for these terms were provided. |
| **Dahan 09** | IFNα-2A | STZ+5FU | 2/32 (6%) v. 3/32 (9%) | NS | 0/32 (0%) v. 0/32 (0%) | NS | "Overall haematological toxicities", "creatininemia", Gd 3-4 (WHO criteria) |
| **Oberg 89** | IFN | STZ+5FU | Not reported | Not reported | Not reported | Not reported |  |
| **Meyer 14** | STZ+Capecitabine | STZ+Capecitabine+Cisplatin | No composite; anaemia 1/40 (3%) vs 0/43 (0%). Neutropaenia 3/40 (8%) vs 0/43 (0%) | Not reported | 4/40 (10%) vs 0/42 (0%) | Not reported |  |

***Table S5****: Grade 3/4 toxicity, % Data for both direct assignment and randomised groups were included*

*^ P-value for overall maximal toxicities*
